# Increasing Community Access to Fresh Fruits and Vegetables: A Case Study of the Farm Fresh Market Pilot Program in Cobb County, Georgia, 2014

**DOI:** 10.5888/pcd13.150442

**Published:** 2016-03-10

**Authors:** Rebecca C. Woodruff, Anne-Marie Coleman, April K. Hermstad, Sally Honeycutt, Jennifer Munoz, Lorna Loh, Agnes F. Brown, Rebecca Shipley, Michelle C. Kegler

**Affiliations:** Author Affiliations: Anne-Marie Coleman, Jennifer Munoz, Agnes F. Brown, Cobb and Douglas Public Health, Marietta, Georgia; April K. Hermstad, Sally Honeycutt, Michelle C. Kegler, Department of Behavioral Sciences and Health Education, Rollins School of Public Health, Emory University, Atlanta, Georgia; Lorna Loh, Rebecca Shipley, McCleskey-East Cobb Family YMCA, Marietta, Georgia.

## Abstract

**Background:**

Ecological models of health suggest that to effectively prevent chronic disease, community food environments must support healthy eating behaviors. However, disparities in access to healthy foods persist in the United States.

**Community Context:**

The Farm Fresh Market (FFM) was a fruit and vegetable market that sold low-cost fresh produce in Cobb County, Georgia in 2014.

**Methods:**

This case study describes the development of the FFM through a community engagement process and presents evaluation results from the project’s pilot implementation. Community engagement strategies included forming a community advisory board, conducting a needs assessment, and contracting with a community-based organization to implement the FFM.

**Outcome:**

In the pilot year, the FFM served an average of 28.7 customers and generated an average of $140.20 in produce sales per market day. Most returning customers lived in the local community and reported a range of socioeconomic backgrounds. Most returning customers strongly agreed that the FFM made it easier (69.0%) and less expensive (79.0%) for them to buy fresh fruits and vegetables, reported that they ate more vegetables (65.0%) and fruit (55.0%) as a result of the FFM, and reported that they were very satisfied with the FFM overall (92.0%).

**Interpretation:**

Results from this community case study underscore the importance of engaging communities in the development of community food environment interventions. Results also suggest that the FFM initiative was a feasible and acceptable way to respond to the community-identified public health priority of increasing access to healthy foods.

## Background

Increasing fruit and vegetable consumption is a public health priority nationally and in Georgia. In the United States, only 23.4% of adults eat fruits and vegetables 5 or more times per day, and only 24.5% of adults in Georgia do so (1). Markedly fewer adults eat fruits and vegetables in accordance with federal dietary guidelines (2).

Ecological models of health suggest that to effectively prevent chronic disease, community food environments must support healthy eating (3). However, substantial research indicates that neighborhoods do not have equal access to retailers of healthy foods (4) and that access to these retailers may be associated with improved dietary outcomes (5–7).

Understanding community-level determinants of dietary behaviors may explain why fruit and vegetable consumption is below recommended levels and indicate opportunities for intervention (3,5,8). Strategies to improve community food environments are a focus of federal health promotion programs (9) and include increasing the number and density of food retailers that sell fresh produce, such as supermarkets (10,11), farmers’ markets (12), or fruit and vegetable vendors (13,14).

Through the Community Transformation Grant (CTG) program, the Centers for Disease Control and Prevention (CDC) funded communities to implement chronic disease prevention interventions, including those focused on healthy eating (15). Cobb and Douglas Public Health (CDPH), a local public health department in Georgia, used CTG funding to develop and pilot the Farm Fresh Market (FFM), a novel community-based intervention to increase access to fresh fruits and vegetables among residents of Cobb County, Georgia. This case study describes the development, implementation, and evaluation of the FFM pilot project.

## Community Context

The FFM took place in the 30168 zip code, a largely unincorporated part of Cobb County, Georgia. Of the 26,365 residents estimated to be living in this area from 2010 through 2014, approximately 29.6% of residents and 42.4% of children were living below the poverty level (16). Although epidemiologic data are not available for the 30168 zip code, county-level estimates indicate that overweight, obesity, and low levels of fruit and vegetable consumption are public health priorities in Cobb County. For example, most county residents are overweight or obese, and only 28.8% of residents report eating 5 or more servings of fruits and vegetables per day (17,18). According to an asset map compiled by CDPH, no supermarkets or grocery stores are located in the 30168 zip code, suggesting that residents have limited access to healthy food options (19).

The community engagement objective for this project was to gather community input on priority public health issues and the resulting FFM initiative in Cobb County, Georgia. The objective of the FFM initiative was to increase access to fresh fruits and vegetables among residents of the 30168 zip code.

## Methods

### Needs assessment

CDPH partnered with Cobb2020, a multisectoral partnership of 27 local organizations and community members that provides strategic planning and guidance to CDPH, to conduct a needs assessment using the Mobilizing for Action through Planning and Partnerships (MAPP) strategic planning process, which led to the development of the FFM. In 2011, CDPH and Cobb2020 surveyed county residents with a listed landline or cellular telephone number. Participation was limited to one randomly sampled adult per household. Most of the 1,244 respondents were middle-aged (mean age, 43.8 y; standard deviation, 16.7 y), female (65.7%), and reported their race/ethnicity as white (75.9%). Respondents ranged in educational attainment and household income; 21.2% reported a high school diploma or GED (general educational development) or less, and 15.1% reported an annual household income of $25,000 or less (18).

A finding from this survey was that most county residents were either overweight (36.0%) or obese (22.3%), and obesity was more prevalent among people from low-income households than among people from high-income households (18). Many respondents also reported that obesity and other chronic diseases are serious health concerns for the county (18).

In 2012, also as part of MAPP, CDPH conducted 6 focus groups with county residents to better understand barriers to and facilitators of health. CDPH provided minigrants to gatekeeper community groups to recruit participants and contracted with a county university to lead the focus groups. Adults who lived in Cobb County were recruited online, through announcements, and during one-on-one meetings (eg, CDPH clients who came in for services). Four focus groups were conducted in English and 2 in Spanish. Of the 58 participants, 76% were women and 43% reported an annual household income of less than $25,000. Approximately 83% of participants in English-language focus groups were African American, and all participants in the Spanish-language focus groups were Hispanic.

In addition to themes about the need for health education, improved access to health care, increased trust in medical providers, reduced barriers to seeking health care, and greater availability of health care services, participants articulated a need for improved access to affordable, healthy food choices (20). Participants also suggested that farmers’ markets might be a strategy to increase access to fresh fruits and vegetables. Cobb2020 and CDPH used MAPP findings to prioritize intervention strategies that address the community-identified priority of obesity and chronic disease prevention by addressing the lack of access to affordable, healthy foods.

In addition to the MAPP activities, CDPH worked with Cobb2020 to identify health-promoting resources in the county, such as fitness and recreation facilities, health care clinics, and food retailers. These resources were geocoded on an interactive online asset map (19). CDPH and Cobb2020 used this map to identify the 30168 zip code as a community with limited access to healthy food options. CDPH and Cobb2020 used results from this asset map, in conjunction with MAPP results that indicated the community’s low socioeconomic status and high prevalence of children living in poverty, to select families with children aged 5 to 12 living in the 30168 zip code as the priority population for the FFM pilot.

### Development and Implementation of the Farm Fresh Market

CDHP awarded a contract to a local YMCA and Cobb2020 partnership member to develop and implement the FFM. In 2014, CDPH and the YMCA assembled a community advisory board, consisting of community members and representatives from local businesses and organizations (eg, representatives from a local community task force and apartment complexes), to help plan the FFM. Some aspects of the FFM were determined by the YMCA on the basis of feasibility (eg, procurement of fruits and vegetables, price point). Other aspects of the FFM were determined with community advisory board input (eg, market locations, hours, promotion).

From May through September 2014, CDPH and the YMCA piloted the FFM as a low-cost fruit and vegetable market at 3 sites in the 30168 zip code. The FFM sites were the parking lots of 2 apartment complexes and one community recreation center. Each site was open once per week from 5:30 PM to 7:30 PM. Each week, YMCA staff and volunteers purchased bulk quantities of 15 different types of fresh fruits and vegetables from a restaurant distributor and packaged them into individual items of approximately equivalent size and value. Examples of items for sale at the market included 2 apples, 1 head of broccoli, and 1 bunch of collard greens. Customers selected 10 of 15 items to purchase for a set price of $5, paid in cash. CTG funds covered the cost of the produce and the FFM staff. The University of Georgia Cooperative Extension provided recipes, nutrition information and cooking demonstrations, health assessment and awareness workshops, and referrals to healthy lifestyle programs.

Before the market opened, CDPH and YMCA mailed English-language promotional postcards to a commercial mailing list of 30168 zip code residents living with children, distributed flyers in English and Spanish, conducted on-site outreach activities at participating apartment complexes, and produced a promotional video of the FFM.

### Evaluation

The evaluation of the FFM pilot program was designed using a collaborative approach: representatives from CDPH, the YMCA, and Emory University determined the evaluation questions and methods and participated in data collection activities. The goal of the evaluation was to provide descriptive information about FFM implementation, reach, use, and impact. Because the purpose of this evaluation was to generate information used primarily for program improvement, the Emory institutional review board (IRB) determined that this project was a nonresearch program evaluation and did not require IRB approval.

Evaluation data sources included a market tracking log and intercept surveys completed by returning market customers. After each market day, YMCA staff recorded the total number of customers and sales using the market tracking log. On 6 market days in September 2014, toward the end of the pilot season, CDPH and Emory University staff approached FFM customers and invited them to complete self-administered surveys. Customers aged 18 or older who had shopped at the market at least once before were eligible to take the survey and receive a voucher for a free bag of FFM produce as an incentive ($5 value). Because FFM impacts were assessed retrospectively, survey participation was limited to returning customers who could report changes in behavior before and after they began shopping at the FFM.

Market reach was assessed by using standard demographic questions and by asking participants whether they live in the 30168 zip code and have any children aged 5 to 12 living at home. Change in perceived access to healthy foods was measured by asking customers to report the extent to which they agreed or disagreed that the FFM made it easier for them and their family to eat a healthy diet or easier or less expensive for them to buy fresh fruits and vegetables. Customers were also asked to report the distance from their homes to the FFM and the distance they traveled from their homes to purchase fresh fruits and vegetables before they began shopping at the FFM. Responses to these questions were compared to assess whether the FFM decreased the distance customers travel to purchase fresh produce. Change in fruit and vegetable consumption was measured by asking participants to indicate whether they eat fewer, the same amount, or more fruits and vegetables at the time of the survey than they did before they began shopping at the FFM. The survey also included questions about frequency of shopping at the FFM and satisfaction with various aspects of the FFM (eg, location, hours, prices).

Of the 103 customers who were invited to take the survey, 102 completed surveys (99.0% response rate). Two surveys were later excluded, because the participants were ineligible (one was shopping for the first time and one had taken the survey before), resulting in a final sample size of 100 participants. Data were analyzed using SAS 9.3 (2012, SAS Institute, Inc) using descriptive statistics. Chi-square tests and Wilcoxon-Mann-Whitney tests were used to assess differences in demographic characteristics and self-reported changes in perceived access to healthy foods and fruit and vegetable consumption based on FFM shopping frequency (≥once/week vs < once/week).

## Outcome

### Farm Fresh Market Implementation

The FFM sold fresh produce in the 30168 zip code during 22 weeks from May through September 2014. During the pilot season, the FFM attracted an average of 28.7 (range, 5–63) customers per market day (Table 1). Sales ranged from $23.00 to $315.00, with a mean of $140.20 per market day. Customer volume and sales varied across sites.

**Table 1:** Summary of Farm Fresh Market Pilot Implementation, Cobb County, Georgia, May–September, 2014^a^

Item	Mean (Standard Deviation)	Range
**Total no. of customers**		
All markets	28.7 (14.57)	5–63
Site 1	38.9 (12.80)	16–63
Site 2	27.5 (13.41)	5–62
Site 3	19.0 (11.23)	5–41
**Weekly sales, $**		
All markets	140.20 (75.93)	23.00–315.00
Site 1	185.83 (71.73)	47.50–315.00
Site 2	136.14 (74.20)	25.00–310.00
Site 3	98.83 (57.05)	23.00–205.00

a One market day was excluded due to missing data.

### Farm Fresh Market Reach and Use

Most survey participants were female (79.0%) and non-Hispanic black (84.0%) (Table 2). The FFM served customers from varied age ranges, income levels, and educational backgrounds; 15.0% of FFM customers reported that they had obtained a high school education or less, and 10.0% reported an annual household income of $10,000 or less. Nineteen percent of customers reported that they would pay with SNAP EBT (Supplemental Nutrition Assistance Program, Electronic Benefit Transfer) cards, and 11.0% indicated that they would pay using WIC (Special Supplemental Nutrition Program for Women, Infants, and Children), if the options were available.

**Table 2:** Demographic and Reach-Related Characteristics of Farm Fresh Market (FFM) Returning Customers, by Frequency of Shopping at the Market, Cobb County, Georgia, May–September, 2014^a^

Demographic Characteristics	All Participants, n (N = 100)	Shops at FFM Once Per Week or More (n = 49)	Shops at FFM Less Than Once Per Week (n = 51)	*P* Value
		n (%)		
**Female sex**	79	37 (75.5)	42 (82.4)	.40
**Age, y**				
18–29	9	3 (6.1)	6 (11.8)	.10
30–39	16	7 (14.3)	9 (17.7)	
40–49	15	7 (14.3)	8 (15.7)	
50–59	28	13 (26.5)	15 (29.4)	
60–69	21	11 (22.5)	10 (19.6)	
≥70	11	8 (16.3)	3 (5.9)	
**Race/ethnicity**				
Non-Hispanic black	84	39 (79.6)	45 (88.2)	.13
Non-Hispanic white	13	9 (18.4)	4 (7.8)	
Hispanic	1	1 (2.0)	0	
Other	2	0	2 (3.9)	
**Employment status**				
Working full-time	44	19 (38.8)	25 (49.0)	.45
Working part-time	3	2 (4.1)	1 (2.0)	
Retired	27	16 (32.7)	11 (21.6)	
Not employed/homemaker/student/on disability	26	12 (24.5)	14 (27.5)	
**Educational attainment**				
High school/GED or less	15	12 (24.5)	3 (5.9)	.11
Some college or technical school	28	11 (22.5)	24 (47.1)	
College graduate	35	12 (24.5)	10 (19.6)	
Postgraduate or professional degree	22	14 (28.6)	14 (27.5)	
**Annual household income, $**				
≤10,000	10	5 (10.2)	5 (9.8)	.89
10,001–25,000	26	15 (30.6)	11 (21.6)	
25,001–50,000	29	11 (22.4)	18 (35.3)	
≥50,001	26	10 (20.4)	16 (31.4)	
Refused	9	8 (16.3)	1 (2.0)	
**Receipt of public assistance**				
Would use SNAP as payment	19	8 (16.3)	11 (21.6)	.50
Would use WIC as payment	11	6 (12.2)	5 (9.8)	.70
**Reach-related characteristics**				
30168 Zip code resident	78	40 (81.6)	38 (74.5)	.39
Has child aged 5–12 at home	29	13 (26.5)	16 (31.4)	.59
30168 Zip code resident with any children aged 5–12 at home	19	6 (12.2)	13 (25.5)	.09

Abbreviations: GED, general educational development; SNAP, Supplemental Nutrition Assistance Program; WIC, Special Supplemental Nutrition Program for Women, Infants, and Children.

a χ^2^ tests were used to assess differences in sex, race/ethnicity (comparing non-Hispanic blacks with non-Hispanic whites), employment status (collapsing those who work full-time or part-time into 1 category), receipt of public assistance, and reach-related characteristics, by frequency of market attendance. Wilcoxon-Mann-Whitney tests were used to assess differences in age, educational attainment, and annual household income by frequency of market attendance.

Most FFM customers (78.0%) reported that they lived in the 30168 zip code, and 29.0% reported having at least one child aged 5 to 12 living at home. Nineteen percent of customers were from the FFM priority population: residents of the 30168 zip code who had at least one child aged 5 to 12 living at home. Approximately half (49.0%) of customers reported that they shop at the FFM once per week or more. There were no significant differences in the demographic or socioeconomic characteristics of participants on the basis of FFM shopping frequency.

### Changes in Perceived Access to Healthy Foods and in Fruit and Vegetable Consumption

Before they began shopping at the FFM, most customers (58.0%) reported that they traveled 1 to 5 miles from home to purchase fresh fruits and vegetables; few customers (22.0%) traveled less than 1 mile from their homes. By contrast, most customers (55.0%) reported that the FFM is located within 1 mile of their homes (data not shown).

Most customers reported that they strongly agree that the FFM made it easier (69.0%) and less expensive (79.0%) for them to buy fresh fruits and vegetables and that the FFM made it easier for them (63.0%) and their families (64.0%) to eat a healthy diet (Table 3). Compared with respondents who shopped at the FFM less than once per week, a larger proportion of respondents who shopped at the FFM once per week or more strongly agreed that the FFM made it easier for them to eat a healthy diet (*P* = .01), easier for their families to eat a healthy diet (*P* = .04), and easier for them to buy fresh fruits and vegetables (*P* = .047).

**Table 3:** Changes in Perceived Access to Healthy Foods and Self-Reported Fruit and Vegetable Consumption Among Returning Farm Fresh Market (FFM) Customers, by Frequency of Shopping at the Market, Cobb County, Georgia, May–September, 2014

Response	All Participants, n (N = 100)	Shops at FFM Once Per Week or More (n = 49)	Shops at FFM Less Than Once Per Week (n = 51)	*P* Value
		n (%)		
**Strongly agree that the FFM made it . . .^a^**				
Less expensive to buy fresh fruits and vegetables	79	41 (83.7)	38 (74.5)	.11
Easier for me to buy fresh fruits and vegetables	69	38 (77.6)	31 (60.8)	.047
Easier for my family to eat a healthy diet	64	36 (73.5)	28 (54.9)	.04
Easier for me to eat a healthy diet	63	37 (75.5)	26 (51.0)	.01
**As a result of shopping at the FFM, I eat more vegetables^b^**	65	33 (67.4)	32 (62.8)	.63
**As a result of shopping at the FFM, I eat more fruit^b^**	55	27 (55.1)	28 (54.9)	.98

a Other response options (somewhat agree, neither agree nor disagree, somewhat disagree, and strongly disagree) were collapsed to create a binary variable.

b Other response options (eating the same amount of fruits or vegetables or fewer fruits and vegetables) were collapsed to create a binary variable.

Most customers reported that they eat more vegetables (65.0%) and more fruit (55.0%) as a result of shopping at the FFM. There were no significant differences in changes in fruit and vegetable consumption on the basis of FFM shopping frequency. 

### Market Use and Customer Satisfaction

Eighty-eight percent of customers reported that they eat most or all of the fruits and vegetables that they purchase at the market (data not shown). Customers reported very high satisfaction levels with the FFM: 92.0% reported that they were very satisfied with the FFM overall, and most reported that they were very satisfied with the price for a bag of fruits and vegetables (86.0%), with the market locations (85.0%) and hours (84.0%), and the selection of vegetables (67.0%) and fruits (60.0%) (data not shown).

## Interpretation

This case study describes the development, implementation, and evaluation of the FFM, a novel community-based initiative to increase access to healthy foods in Cobb County, Georgia. During the pilot season, the FFM was implemented as planned and sold fresh fruits and vegetables for 22 weeks from May through September 2014. It is noteworthy that the total volume of customers served by the FFM was modest (an average of 28.7 total customers per day) and that only 19.0% came from the priority population (30168 zip code residents with children aged 5 to 12 living in the home). CDPH identified this priority population in the CTG proposal development phase, although community partners also suggested that the FFM attempt to reach any 30168 zip code residents affected by health disparities. Evaluation results indicated that the FFM succeeded at attracting residents from the local community (78% lived in the 30168 zip code) from a range of socioeconomic backgrounds.

Evaluation results also suggested that the FFM may have achieved its goals of increasing perceived access to healthy foods, particularly among those who reported shopping at the FFM most frequently, and increasing fruit and vegetable consumption among returning customers. These findings are consistent with evaluations of similar initiatives. For example, 43 returning shoppers at a mobile produce van reported a 0.37-serving increase (95% confidence interval, −0.23 to 1.14) in daily fruit and vegetable consumption from baseline to follow-up (14). Additionally, 61 residents of a low-income, predominately Hispanic community who lived within 1 mile of a newly introduced produce stand reported an increase of 0.43 daily servings of fruits and vegetables (standard deviation, 0.42; *P* = .21) (13). More rigorous outcome evaluations of these initiatives would help public health researchers, practitioners, and policy makers to better understand the potential health-related impacts of this approach (5).

This is among the first studies describing the development of a new retailer of healthy foods introduced into a community to improve access to fruits and vegetables. This initiative used 3 levels of community engagement: 1) a needs assessment, which provided community members with an opportunity to voice priorities and concerns; 2) a community advisory board, which helped identify a low-cost fruits and vegetables market as a method of addressing limited access to healthy food options and to determine the priority population; and 3) contracting with a community-based organization to serve as the implementation lead and get community input on the FFM. Published studies of similar initiatives have provided little information about whether and how community members were engaged (13,14). Practitioners may benefit from more descriptions of engagement strategies they can use to develop food environment interventions in their communities. Cobb2020 is exploring how to sustain the FFM without CTG funding. In 2015, Cobb2020 implemented the FFM at 2 sites. The YMCA continued to operate the FFM and subsidized the staffing and partnered with a local food bank, which donated the fruits and vegetables sold at the market.

Limitations of this evaluation were that market reach and impact were assessed using weak, retrospective measures as part of a cross-sectional survey administered to a convenience sample of returning customers. Although the high response rate of 99.0% is a strength of this evaluation and limits nonresponse bias, this evaluation recruited customers from a nonrandom sample of market days late in the pilot season, and only returning customers were eligible to take the survey. This sampling method may have biased survey results in favor of the market if customers who had less positive experiences with the market stopped shopping there before the evaluation. This evaluation would have been strengthened by the use of a comparison group or longitudinal follow-up with customers recruited throughout the market season. Additionally, the market records used to assess implementation had some missing data and occasionally reported total customer volumes that did not match the number of total sales, given that each bag was uniformly priced at $5.00. 

Despite these limitations, results from this case study underscore the importance of engaging community stakeholders in the development of community food environment interventions and suggest that the FFM was a feasible and acceptable way for CDPH and Cobb2020 to respond to community-identified public health priorities by increasing access to healthy foods.
